# Bullying Victimization, Real and Perceived Physical Fitness, and Self-Perception Profiles in Middle-School Students with Overweight or Obesity

**DOI:** 10.3390/nu15245019

**Published:** 2023-12-06

**Authors:** Giada Ballarin, Francesca Gallè, Lucia Dinacci, Federica Liberti, Fabrizio Liguori, Maria Cristina Nisco, Antonia Cunti, Giuliana Valerio

**Affiliations:** Department of Medical, Movement and Wellbeing Sciences, University of Naples “Parthenope”, 80133 Naples, Italy; giada.ballarin001@studenti.uniparthenope.it (G.B.); francesca.galle@uniparthenope.it (F.G.); lucia.dinacci001@studenti.uniparthenope.it (L.D.); libertifederica7@gmail.com (F.L.); fabrizio.liguori91@gmail.com (F.L.); mcristina.nisco@uniparthenope.it (M.C.N.); antonia.cunti@uniparthenope.it (A.C.)

**Keywords:** body mass index, school, self-perception, physical fitness, victimization

## Abstract

Bullying victimization has been associated with body weight and physical fitness. In order to compare the prevalence of victimization among adolescents with normal weight or overweight/obesity and to evaluate the association between real or perceived physical fitness and self-perception profiles, a sample of 128 middle-school youths (mean age: 12.2 ± 0.8 years, 61% males) was recruited. The body mass index (BMI) Z score was calculated. Physical fitness was assessed by hand grip strength (HGS), long jump (LJ), and a 3 min step test (3MST). Perceived physical fitness, self-perception profiles (social competence, athletic competence, and physical appearance), and experiences of victimization (teasing about physical appearance, verbal offenses, and exclusion from group activities) were explored using validated questionnaires. Eighty youths (62.5%) showed overweight/obesity. Compared to their counterparts, normal-weight subjects showed lower HGS and perceived muscular strength; higher LJ and 3MST performance; higher perceived overall fitness, speed and flexibility, and physical appearance. Teasing and verbal offences were more frequent among students with overweight/obesity. Being teased about physical aspects was positively related with the BMI Z score. Exclusion from groups was positively associated with LJ performance. All the types of victimization investigated were inversely related to perceived social competence. These findings suggest that self-perception may protect one from victimization, regardless of nutritional status.

## 1. Introduction

Episodes of bullying victimization, which refers to the process by which a person is repeatedly and over time exposed to intentional negative actions by their peers, often occur in the school years, affecting social, academic, and psychological aspects of a student’s life [1]. Bullying victimization is commonly classified into traditional forms, which include physical contact (e.g., hitting), verbal harassment (e.g., teasing), exclusion from a group and obscene gestures, and cyberbullying, which involves the use of electronic devices [2,3,4]. From the perspective of bullying prevention, it is important to identify those elements that can put children and adolescents at risk of victimization.

Several children’s characteristics have been shown to be associated with victimization in the school setting. In terms of age, the highest incidence is observed in the transition to middle school (11 years), with a following age-related decline (15 years) [5]. In particular, recent data from the Italian Health Behavior in School-aged Children (HBSC) survey showed a peak in victimization due to traditional bullying among 11-year-old adolescents (16–18%), with a reduction among 15-year-old students (9–10%) [6]. 

As for nutritional status, relationships have been found between bullying and overweight/obesity [7,8]. In childhood and adolescence, weight stigmatization can lead to victimization, teasing, and bullying experiences, which can be direct (e.g., verbal teasing or physical violence) or relational (e.g., social exclusion) [9]. During middle school, weight-based bullying is more severe and frequent for overweight youth than for non-overweight youth, and weight is the most common reason for teasing at school, even more frequent than other reasons for bullying such as sexual orientation and race [9]. Evidence shows that weight-based victimization can affect children’s and adolescents’ emotional, social, academic, and physical wellbeing. In fact, it can compromise the victims’ ability to form supportive social relationships with peers, disengage students from their school environments, and interfere with health behaviors, such as participation in sports or other physical activities. As a consequence, a vicious circle occurs, reinforcing excess weight and leading to poor health outcomes [7,10,11,12,13]. Indeed, victims of weight-based teasing are also more likely to engage in disordered eating behaviors or substance abuse [14,15].

Furthermore, traditional forms of bullying victimization have been found to be associated with subjectively or objectively assessed physical fitness components. In particular, cardiorespiratory fitness was shown to be protective against traditional bullying, even among children and adolescents with overweight and obesity [5,16]. It was argued that youths with an optimal level of CRF may have higher perceived physical ability and perceived physical appearance, which can lead to greater involvement in sport, school, and social activities and a lower likelihood of experiencing bullying [13,17]. It seems, therefore, that youths who show the “fat but fit” phenotype are accepted and may carry a reputation in the social group, which can protect them from bullying [5]. However, this evidence is still scarce and limited to a few countries.

Moreover, recent evidence has shown that even COVID-19 led to an increase in traditional bullying among adolescents [18].

In order to contribute to the research on this subject, in this study, we assessed the prevalence of bullying victimization in a sample of normal-weight students from a middle school in southern Italy compared to their peers with overweight/obesity and evaluated the possible association between bullying victimization, real or perceived physical fitness, and self-perception profiles.

We hypothesized that the following: 1. bullying victimization is related to overweight/obesity; 2. better objective or self-perceived physical fitness can reduce the risk of being bullied; 3. self-perceived competence can reduce the risk of bullying victimization.

## 2. Materials and Methods

This cross-sectional study was performed in two middle schools in Naples (Italy). All children and their parents or legal guardians agreed to sign the written informed consent for all procedures before the enrolment. This study was approved by the Ethical Committee of the University of Campania “Luigi Vanvitelli” (protocol number n. 8383/2023). One hundred and seventy-two adolescents (57% boys, 43% girls) attended the selected schools in Naples, Italy. Of these, 128 (61% boys, 39% girls) gave their consent to participate in the present study.

### 2.1. Anthropometric Measurements

The participants were measured by a skilled operator following standard procedures, with the participants wearing only light clothes and no shoes. Body weight and height were measured to the nearest 0.1 kg and 0.5 cm, respectively, using a medical scale and a portable stadiometer. BMI was calculated as weight/stature^2^ (kg/m^2^) and transformed into BMI Z scores according to the WHO growth reference [19]. Normal weight was defined as a BMI Z score between −2 and +0.99, overweight as a BMI Z score > 1, and obesity as a BMI Z score > 2.

### 2.2. Physical Fitness Assessment

The following physical tasks were assessed according to standard procedures: hand grip strength (HGS), long jump (LJ), and a 3 min step test (3MST).

HGS was measured with a Takei 5401 digital dynamometer to assess the isometric strength of the dominant and non-dominant arms. Participants were instructed to stand upright with their shoulders adducted and neutrally rotated, their elbows fully extended, and their forearms and wrists neutrally positioned during the study. A pre-test was performed to allow participants to be familiar with the instrument. Participants performed three maximal isometric contractions (each lasting 5 s) with each hand, with a 1 min rest between tests. The maximum HGS was finally defined as the highest value of the six attempts. The results were expressed in kilograms [20].

LJ was used to assess lower-body muscle strength [21]. Participants performed a two-foot take-off and landing. The swinging of the arms and flexing of the knees were permitted to provide forward drive. Participants attempted to jump as far as possible, landing on both feet without falling backward. The length was measured to the nearest point of contact on the landing. Two attempts were performed, and the best value was used for analysis. The results were expressed in centimetres.

3MST was used to assess cardiovascular fitness [22]. Participants were asked to continuously step onto and off a box for 3 min. The number of steps was recorded at the end of the test.

### 2.3. Questionnaires

Questionnaires were administered by interview in a separate room by a trained researcher. Participants were asked to report their sports engagement in the previous 6 months (yes/no).

Furthermore, the International Fitness Scale (IFIS), created and validated by Ortega et al. [23], was used to assess the individual perception of physical fitness. The IFIS is composed of questions on a five-point Likert scale (from one (very poor) to five (very good)) focused on five macro-areas of fitness: general fitness, cardiorespiratory fitness, strength, speed–agility, and flexibility. The questions were as follows:

“Please, think about your current level of physical fitness (compared with your friends) and choose the most appropriate answer.”

- “My general fitness is: Very poor (1); Poor (2); Average (3); Good (4); Very Good (5)”.
- “My cardiorespiratory fitness (capacity to do exercise, for instance long running) is: Very poor (1); Poor (2); Average (3); Good (4); Very Good (5)”.
- “My muscular strength is: Very poor (1); Poor (2); Average (3); Good (4); Very Good (5)”.
- “My speed/agility is: Very poor (1); Poor (2); Average (3); Good (4); Very Good (5)”.
- “My flexibility is: Very poor (1); Poor (2); Average (3); Good (4); Very Good (5).”

The self-perception profile for children by Harter was used to assess social competence, athletic competence, and physical appearance [24]. Every domain consisted of six items, with a score of a maximum of 24 points each (from 1 to 4 points for every question). An example of the items is reported below (Table 1).

The Italian version of the “revised Olweus Victim Questionnaire” was used to assess victimization [1]. On the basis of a previous study performed on a large sample of Italian youths [6,25], three specific types of victimization were selected from the questionnaire: two concerned direct verbal victimization (teasing about physical appearance and verbal offenses), and one concerned relational victimization (exclusion from group activities).

The participants were asked to answer the following questions: “how often have you been bullied at school in the past six months?” using a five-point scale (“never involved”, “only once or twice”, “one or two times a month”, “at least once a week”, and “almost every day”). To categorize involvement in victimization, adolescents were classified as victims if they exceeded a cut-off of one or two episodes a month or more in the last six months.

### 2.4. Statistical Analysis

The results were reported as mean ± standard deviation (or standard error) or number and percentage. The Shapiro–Wilks normality test was applied to assess the normality of the data distribution; all the variables exhibited a nonparametric distribution. Differences among groups were assessed using a one-way ANOVA or a general linear model (where data were adjusted for age). For this scope, variables were logarithmically transformed but expressed as untransformed values for clarity of interpretation. The Spearman’s rank correlation test was applied to assess the association between real and perceived physical fitness tests, or self-perception profiles. Categorical variables were compared using a chi-squared linear-by-linear association (Mantel–Haenzel test for trend). A logistic regression analysis was used to estimate the relationship between victimization experiences and the variables of interest. Specifically, several logistic regression models were run, where each victimization item was the dependent variable, and age, sex, BMI Zscore, each physical fitness test or perceived physical fitness macro-area, and self-perception domain were the independent variables.

Statistical significance was pre-determined at *p* < 0.05. The effect size was calculated on the non-adjusted variables according to Cohen, considering a small effect size for d from 0 to 0.20, a medium effect size for d from 0.21 to 0.50, and a large effect size for d > 0.50 [26]. All statistical analyses were performed using the Statistical Package for Social Sciences (SPSS Inc, Chicago, IL, USA) version 28.

## 3. Results

Participants were grouped as normal weight (*n* = 48, 37.5%) and overweight/obesity (*n* = 80, 62.5%, of which *n* = 39 overweight and *n* = 41 adolescents with obesity). General characteristics, sports participation, and outcomes related to physical fitness; perceived physical fitness, self-perception profiles, and victimization experiences in the total sample; and subgroups stratified according to BMI status or sex are described in Table 2.

No differences were found in sport practice when the data were divided by BMI groups, while a significant difference was found by sex.

As far as the performance in physical fitness tests is concerned, HGS was higher, while LJ was lower in the group with overweight/obesity. No difference was found with regard to perceived cardiorespiratory fitness between BMI groups. As expected, physical fitness tests were greater in boys vs. girls (*p* < 0.05 for all). Perceived muscular strength was higher in the overweight/obesity group compared to the normal weight counterpart, and in boys vs. girls, while the remaining macro-areas were greater in the normal-weight group compared to the overweight/obesity group (*p* < 0.05; no significant sex-related differences).

The domains of self-perception profiles did not differ between groups except for the perceived physical appearance, which was higher in the normal-weight group compared to overweight/obesity. As far as sex-related differences were concerned, all domains of self-perception were higher in boys vs. girls, except for social competence.

The prevalence of victimization experiences ranged between 8.7% and 21.4% in the whole sample. Teasing about physical appearance and bad words were significantly more prevalent in the overweight/obesity group versus the normal-weight group. Similarly, exclusion from group activities was higher in the overweight/obesity group, but this difference did not reach statistical significance. No statistical differences were found when boys vs. girls were considered.

Table 3 shows the results of the correlation analyses performed between objectively measured fitness, perceived fitness, and self-perception domains.

A significant correlation was found between the LJ performance and perception of overall fitness, muscular strength, speed, and physical self-perception domains. HGS significantly correlated with perceived muscular strength.

The results of the logistic regression analyses are shown in Table 4.

The BMI Z score was significantly associated with teasing for physical appearance. As for physical fitness domains, the long jump performance was positively associated with exclusion from the group. Among the macro-areas of perceived physical fitness, the OR of being teased for physical aspect and excluded from group was negatively associated with the overall fitness perception, while the OR of receiving verbal offences was negatively associated with perception of flexibility. Any kind of bullying victimization was negatively associated with social self-perception profile, while the OR of being excluded from group and verbal offences was negatively associated with sport self-perception profile.

## 4. Discussion

This study investigated the prevalence of bullying victimization episodes in a sample of middle-school students with normal weight or overweight/obesity and the possible association between victimization, objectively measured or perceived physical fitness, and self-perception profiles.

Similar rates of teasing about physical appearance and verbal offences were reported by participants (roughly 20%), with a significantly higher frequency in children and adolescents with overweight/obesity compared to those of normal weight. Exclusion from group activities was less frequently reported (roughly 9%), which was also more frequent in the overweight/obesity group. Our findings confirm that weight-based teasing and verbal offences are common experiences reported by youths, particularly for those with a higher body weight [4,5,9,25,27]. These experiences may have negative consequences since they are associated with psychological distress and may predict obesity and adverse eating behaviours into adulthood [28,29,30], given the increase in obesity prevalence observed worldwide during the COVID-19 pandemic [31].

Weight-related differences in victimization are in line with previous works within the literature [6,7,8,9,10] and agree with the results of a previous survey aimed at assessing bullying and victimization among 6–14-year-aged subjects throughout the Italian territory, even though participants with overweight/obesity from our sample showed slightly lower prevalence rates of the examined bullying victimization domains [25].

Sports participation involved about 57% of the sample, which is a proportion lower than the 65.6% reported by the Italian institute of Statistic for the Italian population aged 11–14 years and did not differ between BMI groups [32]. Although we did not assess the family socio-economic level, the two schools are situated in an underprivileged area of the city, which could explain the low sports engagement of participants.

With regard to physical fitness measurements, our results show that HGS was higher in adolescents with overweight/obesity, in line with previous papers in the literature, which describe higher performance in absolute values of HGS (but lower when standardized for weight) [33,34,35]. On the other hand, as expected, LJ was lower in the overweight/obesity group compared to the normal-weight group (112.7 cm vs. 126.6 cm), confirming the previous findings on this topic [36]. Surprisingly, no statistical differences emerged for cardiorespiratory fitness (STEP). Participants with overweight/obesity reported lower scores in perceived overall physical fitness, speed, and flexibility, and performed worse than the normal-weight group in lower limb strength. On the other hand, they reported higher scores in perceived muscular strength and also performed better than the normal-weight group in upper limb strength. Our findings are in line with previous national and international research that also used the IFIS scale in youth [23,37]. It has been demonstrated that IFIS is a valid tool for ranking adolescents according to their objectively measured physical fitness levels [23]. It is worth mentioning that we found a significant relationship between perceived muscular strength and both upper and lower limb strength, measured following the same protocol used by Ortega et al. [23]. On the contrary, we did not find any correlation between the perceived and measured cardiorespiratory fitness; this could be related to the type of test used to assess cardiorespiratory fitness since we had to choose the 3M-ST instead of the 20 m shuttle run test due to the characteristics of the school setting. Lastly, we found that perceived overall fitness, speed, and flexibility were also correlated with LJ, suggesting that this test might help in the prescription and monitoring of a tailored exercise program.

As for the self-perception domains, the two BMI groups differed only in their relationship with perceived physical appearance. This result is in line with the literature, which testifies to a relationship between greater BMI and body dissatisfaction [38].

Furthermore, in our sample, the regression analyses highlighted an inverse relationship between perceived physical fitness and skills and being a bullying victim, as reported previously [16]. However, these results do not match those related to the objective physical fitness, which surprisingly was, at least for the leg strength component, associated with exclusion from groups. In contrast with this, the study by Benı’tez-Sillero et al. showed that better cardiorespiratory cardiovascular endurance, as measured by the shuttle run test, was inversely related to being a victim of bullying, while muscular strength, assessed by the long jump, 30 s sit-ups, and manual dynamometry tests, was positively related with being an aggressor [39].

Interestingly, in our study, exclusion from the group was related to long jump performance, which in turn was positively related, as found in the correlation analyses, to several domains of perceived fitness and skills. Therefore, a discrepancy between youths’ perceptions of their own characteristics and their real physical conditions emerged from these results.

Nevertheless, social competence, which was not correlated with any of the objectively measured fitness domains, was found to be inversely associated with all the forms of bullying victimization considered in this study, which suggests that bullied youths may encounter difficulties in building and maintaining relationships with peers. Furthermore, the various forms of victimizations were associated negatively with a better perceived sense of overall fitness or a better self-perception of social or sport competence.

When interpreting the results of this study, some considerations about its limitations should be recognized. First, the sample was composed those students whose parents provided consent to participate in the study, and its composition may not reflect reality. In fact, it showed a higher prevalence of boys compared to the eligible sample. Second, the prevalence of overweight and obesity in the sample was higher than that reported in the paediatric population living in the same urban area in 2019 (62.5% vs. 47.2%, respectively) [40]. It may also be feasible that the prevalence of overweight/obesity was also increased in the whole eligible school population as an effect of the SARS-CoV-19 epidemic [41,42]. Since it was not possible to obtain anthropometric or demographic information about the students who did not take part in the study, we cannot ascertain if a selection bias might have occurred [43]. Therefore, the results cannot be generalized to the whole population.

However, the strengths of this study are represented by the objective assessment of participants’ anthropometric and fitness characteristics and by the simultaneous assessment and comparison of both objective and perceived fitness-related parameters. Plus, we assessed physical fitness, perceived fitness, bullying experience, and self-perception with standardized methods and validated questionnaires and performed a comprehensive analysis of all these variables. Therefore, our investigation can be considered preliminary to future larger studies in this field.

## 5. Conclusions

The findings of this study underline the association between nutritional status and bullying victimization in adolescence and highlight the role that self-perception, more than actual competence, may play in protecting against bullying.

Given the negative consequences and effects of youth victimization on their health-related quality of life, the awareness of parents, teachers, trainers, and health professionals about weight-based victimization should be enhanced. Promoting physical activity and increasing health-related physical fitness may be useful to protect youths from bullying experiences, thus reinforcing their social competence and self-esteem. School programs of physical education should consider the impairment of motor performance in adolescents with OW or OB and offer activities adapted to low physical fitness in order to foster self-esteem and self-perception. Our preliminary findings highlight the need for future studies to better define the relationship between physical fitness and bullying roles in children of school age.

## Figures and Tables

**Table 1:** Example of the items included in the self-perception profile for children by Harter.

Really True for Me	Sort of True for Me				Sort of True for Me	Really True for Me
1	2	Some kids don’t think that making a lot of friends is all that important	BUT	Other kids think that making a lot of friends is important to how they feel as a person	3	4

**Table 2:** General characteristics, lifestyle behaviors, and bullying in the total sample and subgroups stratified according to BMI status or sex.

	Total Sample (*n* = 128)	Normal-Weight (*n* = 48)	Overweight/Obesity (*n* = 80)	*p*	Boys (*n* = 78)	Girls (*n* = 50)	*p*
Age (years) mean ± SD	12.2 ± 0.8	12.2 ± 0.8	12.3 ± 0.9	0.666	12.4 ± 1.0	12.3 ± 0.8	0.653
Sex Boys *n* (%)	78 (61)	27 (56)	51 (64)	0.404			
Overweight/obesity *n* (%)	-	-	-		51(65.4)	29(58.0)	0.256
Sport practice *n* (%)	72 (57.1)	27 (57.4)	45 (57.0)	0.958	52 (68.4)	20 (40.0)	<0.001
Handgrip strength (kg) ^a^ mean ± SD	22.0 ± 5.9	20.2 ± 0.6	23.3 ± 0.5	<0.001	23.1 ± 0.5	20.7 ± 0.6	0.004
Long jump (cm) ^a^ mean ± SD	118.3 ± 25.3	126.6 ± 3.1	112.7 ± 2.5	0.001	126.5 ± 2.6	105.3 ± 3.2	<0.001
Step test (steps) ^a^ mean ± SD	100.3 ± 23.5	103.5 ± 3.2	96.5 ± 2.5	0.088	104.2 ± 2.5	91.1 ± 3.2	0.002
Perceived physical fitness (IFIS) *							
Overall fitness mean ± SD	3.2 ± 1.0	3.5 ± 0.8	3.1 ± 1.0	0.024 ^α^	3.4 ± 1.0	3.0 ± 0.9	0.062
Muscular strength mean ± SD	3.3 ± 1.0	3.0 ± 1.0	3.5 ± 1.0	0.008 ^β^	3.5 ± 1.0	2.9 ± 1.1	<0.001 ^β^
Cardiorespiratory fitness mean ± SD	3.5 ± 1.1	3.7 ± 1.1	3.4 ± 1.1	0.141	3.6 ± 1.1	3.3 ± 1.1	0.144
Speed mean ± SD	3.7 ± 1.1	4.2 ± 0.9	3.3 ± 1.1	<0.001 ^γ^	3.8 ± 1.1	3.5 ± 1.1	0.150
Flexibility mean ± SD	3.2 ± 1.1	3.5 ± 1.1	3.0 ± 1.0	0.019 ^α^	3.3 ± 1.0	3.2 ± 1.2	0.599
Self-perception profile							
Social competence mean ± SD	3.0 ± 0.7	3.0 ± 0.6	2.9 ± 0.7	0.739	2.8 ± 0.7	3.0 ± 0.7	0.101
Athletic competence mean ± SD	2.5 ± 0.7	2.6 ± 0.7	2.5 ± 0.7	0.195	2.7 ± 0.6	2.3 ± 0.7	0.004 ^β^
Physical appearance mean ± SD	2.6 ± 0.9	2.9 ± 0.8	2.4 ± 0.9	0.004 ^β^	2.8 ± 0.8	2.3 ± 0.9	0.009 ^α^
Bullying victimization experiences							
Teasing about physical appearance *n* (%)	27 (21.4)	5 (18.5)	22 (81.5)	0.023	17 (22.4)	10 (20.0)	0.466
Verbal offences *n* (%)	24 (19.2)	5 (20.8)	19 (79.2)	0.046	12 (16.0)	12 (24.0)	0.189
Exclusion from group *n* (%)	11 (8.7)	2 (18.2)	9 (81.8)	0.170	6 (7.9)	5 (10.0)	0.458

^a^ = mean adjusted for age and sex ± standard error (BMI groups) and adjusted for age (sex differences). * Individual data are expressed as mean ± SD of the 5-point Likert scale. ^α^ small effect size; ^β^ moderate effect size; ^γ^ large effect size.

**Table 3:** Spearman rho correlations between objective physical fitness, perceived physical fitness, and self-perception profile variables.

	Handgrip Strength	Long Jump	Step Test
Perceived physical fitness			
Overall fitness	0.030	0.209 *	0.011
Cardiorespiratory fitness	−0.007	0.173	0.058
Muscular strength	0.332 *	0.211 *	0.120
Speed	−0.111	0.281 *	0.146
Flexibility	−0.024	0.026	−0.107
Self-perception profile			
Social competence	0.017	0.051	−0.022
Athletic competence	0.104	0.288 **	0.032
Physical appearance	−0.040	0.271 **	0.095

* = *p* < 0.05 for each coefficient ** = *p* < 0.001.

**Table 4:** Results of the logistic regression analyses performed considering victimization experiences as outcomes.

	Teasing for Physical Aspect OR (95% C.I.)	Verbal Offences OR (95% C.I.)	Exclusion from Group OR (95% C.I.)
General characteristics			
Sex (reference girls)	1.080 (0.438; 2.658)	0.553 (0.220; 1.392)	0.721 (0.204; 2.541)
Age	0.836 (0.517; 1.351)	0.724 (0.426; 1.230)	0.848 (0.421; 1.705)
BMI status (reference normal weight)	3.263 * (1.137; 9.359)	2.912 (0.991; 8.555)	3.001 (0.616; 14.625)
Physical fitness			
Handgrip strength	0.946 (0.844; 1.059)	1.012 (0.899; 1.140)	1.058 (0.906; 1.235)
Long jump	0.994 (0.972; 1.017)	1.016 (0.989; 1.043)	1.038 * (1.002; 1.076)
Step test	1.001 (0.981; 1.022)	1.004 (0.982; 1.026)	0.301 (0.983; 1.015)
Perceived physical fitness			
Overall fitness	0.461 * (0.275; 0.772)	0.757 (0.459; 1.249)	0.449 * (0.218; 0.924)
Cardiorespiratory fitness	1.196 (0.782; 1.829)	0.859 (0.556; 1.326)	0.834 (0.465; 1.496)
Muscular strength	1.062 (0.656; 1.719)	0.869 (0.532; 1.420)	0.585 (0.291; 1.178)
Speed–agility	0.982 (0.642; 1.504)	1.362 (0.884; 2.198)	0.719 (0.393; 1.313)
Flexibility	0.869 (0.568; 1.329)	0.597 * (0.378; 0.942)	0.969 (0.529; 1.775)
Self-perception profile			
Social competence	0.431 * (0.227; 0.820)	0.229 * (0.109; 0.481)	0.198 * (0.072; 0.544)
Athletic competence	0.534 (0.256; 1.112)	0.388 * (0.174; 0.868)	0.189 * (0.054; 0.663)
Physical appearance	0.567 (0.319; 1.009)	0.546 (0.298; 1.000)	0.494 (0.209; 1.172)

Sex, age and BMI status are included in all models. * = *p* < 0.05 for each coefficient. OR = odds ratio; C.I. = confidence interval.

## Data Availability

The data presented in this study are available on request from the corresponding author.

## References

[1] Olweus D., Huesmann L.R. (1994). Bullying at School. Aggressive Behavior.

[2] Waasdorp T.E., Bradshaw C.P. (2015). The Overlap between Cyberbullying and Traditional Bullying. J. Adolesc. Health.

[3] Sánchez V., Muñoz-Fernández N., Vega-Gea E. (2017). Peer Sexual Cybervictimization in Adolescents: Development and Validation of a Scale. Int. J. Clin. Health Psychol..

[4] Trivedi M., Frisard C., Crawford S., Bram J., Geller A.C., Pbert L. (2022). Impact of COVID-19 on Childhood Obesity: Data from a Paediatric Weight Management Trial. Pediatr. Obes..

[5] DeVoe J.F., Kaffenberger S., Chandler K., US Department of Education (2005). Student Reports of Bullying: Results from the 2001 School Crime Supplement to the National Crime Victimization Survey: (428692005-001) 2005.

[6] Bullismo-Cyberbullismo-2022.pdf. https://www.epicentro.iss.it/hbsc/pdf/temi2022/bullismo-cyberbullismo-2022.pdf.

[7] Faith M.S., Leone M.A., Ayers T.S., Heo M., Pietrobelli A. (2002). Weight Criticism during Physical Activity, Coping Skills, and Reported Physical Activity in Children. Pediatrics.

[8] Jones D.C., Newman J.B., Bautista S. (2005). A Three-Factor Model of Teasing: The Influence of Friendship, Gender, and Topic on Expected Emotional Reactions to Teasing during Early Adolescence. Soc. Dev..

[9] Puhl R.M., King K.M. (2013). Weight Discrimination and Bullying. Best Pract. Res. Clin. Endocrinol. Metab..

[10] Eisenberg M.E., Neumark-Sztainer D., Story M. (2003). Associations of Weight-Based Teasing and Emotional Well-Being among Adolescents. Arch. Pediatr. Adolesc. Med..

[11] Storch E.A., Milsom V.A., Debraganza N., Lewin A.B., Geffken G.R., Silverstein J.H. (2007). Peer Victimization, Psychosocial Adjustment, and Physical Activity in Overweight and at-Risk-for-Overweight Youth. J. Pediatr. Psychol..

[12] Gray W.N., Janicke D.M., Ingerski L.M., Silverstein J.H. (2008). The Impact of Peer Victimization, Parent Distress and Child Depression on Barrier Formation and Physical Activity in Overweight Youth. J. Dev. Behav. Pediatr..

[13] Merrill R.M., Hanson C.L. (2016). Risk and Protective Factors Associated with Being Bullied on School Property Compared with Cyberbullied. BMC Public Health.

[14] Libbey H.P., Story M.T., Neumark-Sztainer D.R., Boutelle K.N. (2008). Teasing, Disordered Eating Behaviors, and Psychological Morbidities among Overweight Adolescents. Obesity.

[15] Farhat T., Iannotti R.J., Simons-Morton B. (2010). Overweight, Obesity, Youth, and Health-Risk Behaviors. Am. J. Prev. Med..

[16] Wilkins-Shurmer A., O’Callaghan M.J., Najman J.M., Bor W., Williams G.M., Anderson M.J. (2003). Association of Bullying with Adolescent Health-Related Quality of Life. J. Paediatr. Child Health.

[17] Strong W.B., Malina R.M., Blimkie C.J.R., Daniels S.R., Dishman R.K., Gutin B., Hergenroeder A.C., Must A., Nixon P.A., Pivarnik J.M. (2005). Evidence Based Physical Activity for School-Age Youth. J. Pediatr..

[18] Da Q., Huang J., Peng Z., Chen Y., Li L. (2023). Did the Prevalence of Traditional School Bullying Increase after COVID-19? Evidence from a Two-Stage Cross-Sectional Study before and during COVID-19 Pandemic. Child Abus. Negl..

[19] de Onis M., Onyango A.W., Borghi E., Siyam A., Nishida C., Siekmann J. (2007). Development of a WHO Growth Reference for School-Aged Children and Adolescents. Bull. World Health Organ..

[20] Roberts H.C., Denison H.J., Martin H.J., Patel H.P., Syddall H., Cooper C., Sayer A.A. (2011). A Review of the Measurement of Grip Strength in Clinical and Epidemiological Studies: Towards a Standardised Approach. Age Ageing.

[21] Marques A., Henriques-Neto D., Peralta M., Martins J., Gomes F., Popovic S., Masanovic B., Demetriou Y., Schlund A., Ihle A. (2021). Field-Based Health-Related Physical Fitness Tests in Children and Adolescents: A Systematic Review. Front. Pediatr..

[22] Iturain Barrón A., Quintana Riera S., Reychler G. (2021). The 3 Minute Step Test Is a Validated Field Test to Evaluate the Functional Exercise Capacity in Children Aged 6 to 12. Respir. Med. Res..

[23] Ortega F.B., Ruiz J.R., España-Romero V., Vicente-Rodriguez G., Martínez-Gómez D., Manios Y., Béghin L., Molnar D., Widhalm K., Moreno L.A. (2011). The International Fitness Scale (IFIS): Usefulness of Self-Reported Fitness in Youth. Int. J. Epidemiol..

[24] Harter S. (2017). Self-Perception Profile for Adolescents 2017.

[25] Bacchini D., Licenziati M.R., Garrasi A., Corciulo N., Driul D., Tanas R., Fiumani P.M., Pietro E.D., Pesce S., Crinò A. (2015). Bullying and Victimization in Overweight and Obese Outpatient Children and Adolescents: An Italian Multicentric Study. PLoS ONE.

[26] Cohen J. (1992). Quantitative Methods in Psychology. Psychol. Bull..

[27] Xiang G.-X., Li H., Gan X., Qin K.-N., Jin X., Wang P.-Y. (2022). School Resources, Self-Control and Problem Behaviors in Chinese Adolescents: A Longitudinal Study in the Post-Pandemic Era. Curr. Psychol..

[28] Blanco M., Solano S., Alcántara A.I., Parks M., Román F.J., Sepúlveda A.R. (2020). Psychological Well-Being and Weight-Related Teasing in Childhood Obesity: A Case-Control Study. Eat. Weight Disord..

[29] Puhl R.M., Wall M.M., Chen C., Austin S.B., Eisenberg M.E., Neumark-Sztainer D. (2017). Experiences of Weight Teasing in Adolescence and Weight-Related Outcomes in Adulthood: A 15-Year Longitudinal Study. Prev. Med..

[30] Puhl R.M., Lessard L.M. (2020). Weight Stigma in Youth: Prevalence, Consequences, and Considerations for Clinical Practice. Curr. Obes. Rep..

[31] Anderson L.N., Yoshida-Montezuma Y., Dewart N., Jalil E., Khattar J., De Rubeis V., Carsley S., Griffith L.E., Mbuagbaw L. (2023). Obesity and Weight Change during the COVID-19 Pandemic in Children and Adults: A Systematic Review and Meta-Analysis. Obes. Rev..

[32] Istat.it Vita Quotidiana e Opinione Dei Cittadini. https://www.istat.it/it/vita-quotidiana-opinione-cittadini?classificazioni.

[33] Palacio-Agüero A., Díaz-Torrente X., Quintiliano Scarpelli Dourado D. (2020). Relative Handgrip Strength, Nutritional Status and Abdominal Obesity in Chilean Adolescents. PLoS ONE.

[34] Cossio-Bolaños M., Vidal-Espinoza R., Albornoz C.U., Fuentes-Lopez J., Sánchez-Macedo L., Andruske C.L., Sulla-Torres J., Campos R.G. (2022). Relationship between the Body Mass Index and the Ponderal Index with Physical Fitness in Adolescent Students. BMC Pediatr..

[35] Mendoza-Muñoz M., Adsuar J.C., Pérez-Gómez J., Muñoz-Bermejo L., Garcia-Gordillo M.Á., Carlos-Vivas J. (2020). Influence of Body Composition on Physical Fitness in Adolescents. Medicina.

[36] Dumith S.C., Ramires V.V., Souza M.A., Moraes D.S., Petry F.G., Oliveira E.S., Ramires S.V., Hallal P.C. (2010). Overweight/Obesity and Physical Fitness Among Children and Adolescents. J. Phys. Act. Health.

[37] Vandoni M., Lovecchio N., Carnevale Pellino V., Codella R., Fabiano V., Rossi V., Zuccotti G.V., Calcaterra V. (2021). Self-Reported Physical Fitness in Children and Adolescents with Obesity: A Cross-Sectional Analysis on the Level of Alignment with Multiple Adiposity Indexes. Children.

[38] Voelker D.K., Reel J.J., Greenleaf C. (2015). Weight Status and Body Image Perceptions in Adolescents: Current Perspectives. Adolesc. Health Med. Ther..

[39] Benítez-Sillero J.d.D., Corredor-Corredor D., Ortega-Ruiz R., Córdoba-Alcaide F. (2021). Behaviours Involved in the Role of Victim and Aggressor in Bullying: Relationship with Physical Fitness in Adolescents. PLoS ONE.

[40] Report-Aziendale-2019_report-Aziendale-2019-Asl-Na1-Centro.pdf. https://www.epicentro.iss.it/okkioallasalute/report-aziendale-2019/report-aziendale-2019_report-aziendale-2019-asl-Na1-centro.pdf.

[41] Lartey S.T., Jayawardene W.P., Dickinson S.L., Chen X., Gletsu-Miller N., Lohrmann D.K. (2023). Evaluation of Unintended Consequences of COVID-19 Pandemic Restrictions and Obesity Prevalence Among Youths. JAMA Netw. Open.

[42] Capra M.E., Stanyevic B., Giudice A., Monopoli D., Decarolis N.M., Esposito S., Biasucci G. (2022). The Effects of COVID-19 Pandemic and Lockdown on Pediatric Nutritional and Metabolic Diseases: A Narrative Review. Nutrients.

[43] Cheung K.L., Ten Klooster P.M., Smit C., de Vries H., Pieterse M.E. (2017). The Impact of Non-Response Bias Due to Sampling in Public Health Studies: A Comparison of Voluntary versus Mandatory Recruitment in a Dutch National Survey on Adolescent Health. BMC Public Health.

